# Testicular cancer mortality in Latin America and the Caribbean: trend analysis from 1997 to 2019

**DOI:** 10.1186/s12885-023-11511-z

**Published:** 2023-10-27

**Authors:** Yuleizy Crispin-Rios, Mariafe Faura-Gonzales, J. Smith Torres-Roman, Carlos Quispe-Vicuña, Uriel S. Franco-Jimenez, Bryan Valcarcel, Andreas Stang, Katherine A. McGlynn

**Affiliations:** 1 Cancer Research Networking, Universidad Cientifica del Sur, Lima, Peru; 2 Latin American Network for Cancer Research (LAN–CANCER), Lima, Peru; 3 Sociedad Científica San Fernando, Universidad Nacional Mayor de San Marcos, Lima, Peru; 4 Institut Für Medizinische InformatikBiometrie Und EpidemiologieUniversitätsklinikum Essen, Essen, Germany; 5 School of Public Health, Boston University, Boston, Mass USA; 6 Division of Cancer Epidemiology and Genetics, National Cancer Institute, Rockville, MD USA

**Keywords:** Testicular neoplasms, Latin America, Caribbean Region, Mortality

## Abstract

**Background:**

In the last decades, an increasing incidence of testicular cancer has been observed in several countries worldwide. Although mortality rates have been variable in many countries, little information is available from Latin America and the Caribbean (LAC). Therefore, we examined mortality trends of testicular cancer in the last two decades.

**Methods:**

Age-standardized mortality rates (ASMR) of testicular cancer per 100,000 men-years were estimated using the World Health Organization mortality database from 1997 to 2019. We examined the mortality trends and computed annual percent change (APC) for all ages and the following age groups, 15–29, 30–44, 15–44, and ≥ 45 years.

**Results:**

Ten countries had mortality rates greater than 0.43 per 100,000 men, with the highest rates for Chile, Mexico, and Argentina. Significant increases in mortality rates were observed in Argentina, Brazil Colombia, and Mexico in all ages, and < 45 years, while Colombia, Ecuador, Mexico, and Peru reported significant downward trends in males aged ≥ 45 years. Only Chile showed significant decreases for all ages and age groups studied.

**Conclusion:**

Mortality by testicular cancer increased among LAC countries in males of all ages and across age groups. A reduction in mortality rates was observed only in Chilean males of all ages and in men ≥ 45 years in several countries. Strengthening of early detection among symptomatic males may decrease the mortality by this neoplasm.

**Supplementary Information:**

The online version contains supplementary material available at 10.1186/s12885-023-11511-z.

## Introduction

Testicular cancer (TC) is relatively rare, but it is the most frequently diagnosed neoplasm among young males aged 20 to 44 years [1], especially in developed countries [2–4]. In 2020, GLOBOCAN reported 74,000 new cases (0.4% of all cancers), representing 1.8 cases per 100,000 men-years, and nearly 9,300 deaths (0.1% of all cancers), representing 0.2 deaths per 100,000 men-years worldwide, with North America and Europe having the highest burden [5, 6]. The incidence of testicular cancer is growing worldwide with variable mortality [2, 3, 7].

The great majority of TCs are testicular germ cell tumors. The risk factors for developing germ cell tumors are not well described, although they are known to be associated with other male reproductive disorders including cryptorchidism [8–12], a family history of testicular cancer [10, 12], hypospadias [13], and a previous diagnosis of malignancy in the contralateral testicle [8, 9, 11]. While other factors have been reported to be associated with increased risk in some studies, none have a strong, reproducible association with TC [14–20].

Although the incidence of TC has increased in recent decades, especially in Northern European countries [2, 3], low mortality rates have been reported in these countries due to highly effective therapies [4]. Some Latin American and Caribbean (LAC) countries have also reported increases in the incidence of TC [21], however, mortality by this cancer has not been comprehensively studied in all LAC countries or over a period longer than 20 years. Therefore, we aimed to assess TC mortality trends, overall and by age group, in LAC countries between 1997 and 2019.

## Material and methods

### Data source

TC deaths were obtained from the World Health Organization (WHO) Mortality Database for the period between 1997 (the first year available) and 2019 (the last year available) [22]. The cause-of-death statistics are from country civil registration systems which are compiled by the national authorities and submitted to the WHO each year. These data are official national statistics transmitted to the WHO by the competent authorities of the countries covered. Each member state reports population data together with its mortality data for the population covered by the death registration system, according to the standards of the International Classification of Diseases (ICD) 10th revision (ICD-10). Only data from countries reporting duly coded data according to the ICD were included in the present study. Data were available for the following LAC countries: Argentina, Brazil, Chile, Colombia, Costa Rica, Cuba, Ecuador, Guatemala, Mexico, Nicaragua, Panama, Paraguay, Peru, Puerto Rico, Uruguay, and Venezuela. We identified TC deaths using the ICD-10 (C62) [23]. Mortality rates were analyzed by age group and calendar year. Estimates of the population for each country were obtained from the World Population Prospects 2022 [24].

### Statistical analysis

The age-standardized mortality rates (ASMRs) for TC from 1997 to 2019 were calculated per 100,000 men-years using the SEGI world standard population [25]. We analyzed trends in TC mortality for all ages and for the following age groups: 15–29 years, 30–44 years, ≥ 45 years. In addition to the main analyses, we analyzed the age group between 15–44 years because these are the ages with the highest incidence of TC (Supplementary 1). We calculated the average TC mortality rates (all ages combined) for the last 5 years for the LAC countries, with those exceeding 0.43 deaths being considered as high rates, as they were the highest rates estimated by GLOBOCAN 2020.

Joinpoint regression analysis was performed to analyze the mortality trends using the Joinpoint Regression Program software (National Cancer Institute, Bethesda, Maryland, USA), Version 4.6.0, 2017 [26]. Joinpoints given by the program were identified and the annual percentage change (APC) and corresponding 95% confidence intervals (95%CI) were estimated for each country. APCs were considered statistically significant with a *p*-value < 0.05. We calculated the average APC (AAPC) for countries that had 2 or more attachment joinpoints. In the joinpoint analysis, the program chooses the points of best fit (joinpoints), where the rate changes significantly. The analysis starts with the minimum number of joinpoints (zero, which is the straight line), and checks whether one or more joinpoints (up to three) are significant and should be added to the model. Each significant joinpoint that indicates a change in slope (if any) is retained in the final model. Mortality trend analyses could not be performed for countries with low death counts in any given year or for the age group 0–14. The significance levels utilized are based on the Monte Carlo permutation method and for the calculation of the APC we used the logarithm of the ratio [27].

## Results

**Fig. 1:**
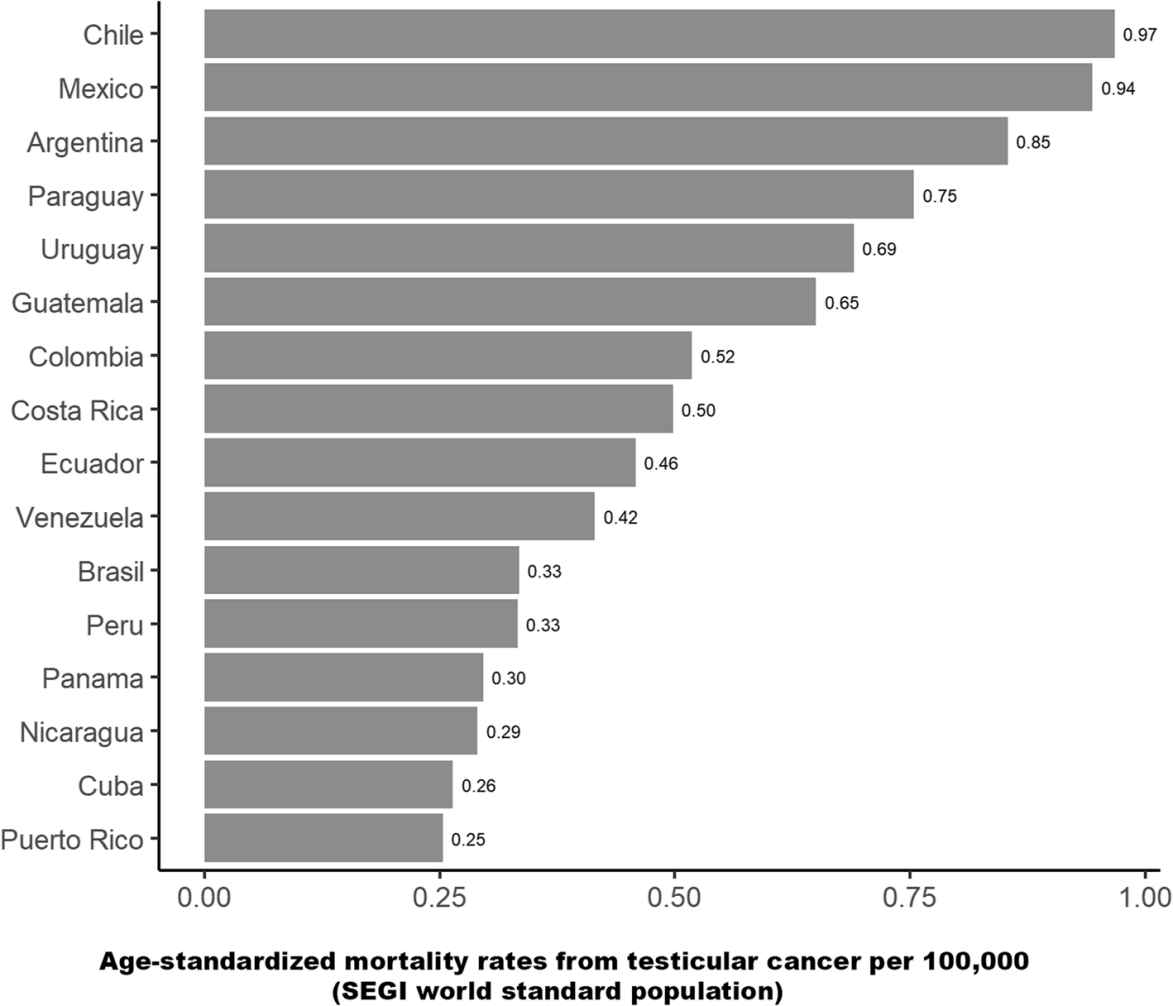
Average age-standardized (Segi world standard population) testicular cancer mortality rates per 100,000 between 2015 and 2019* in Latin America and the Caribbean. *Data from 2018 for Chile and Peru, 2017 for Puerto Rico, 2016 for Venezuela

Figure 1 shows the average age-standardized TC mortality rates per 100,000 between 2015 and 2019 in LAC. Ten countries reported mortality rates higher than 0.43 deaths per 100,000 men, with the highest rates in Chile (0.97), Mexico (0.94), and Argentina (0.85).

**Fig. 2:**
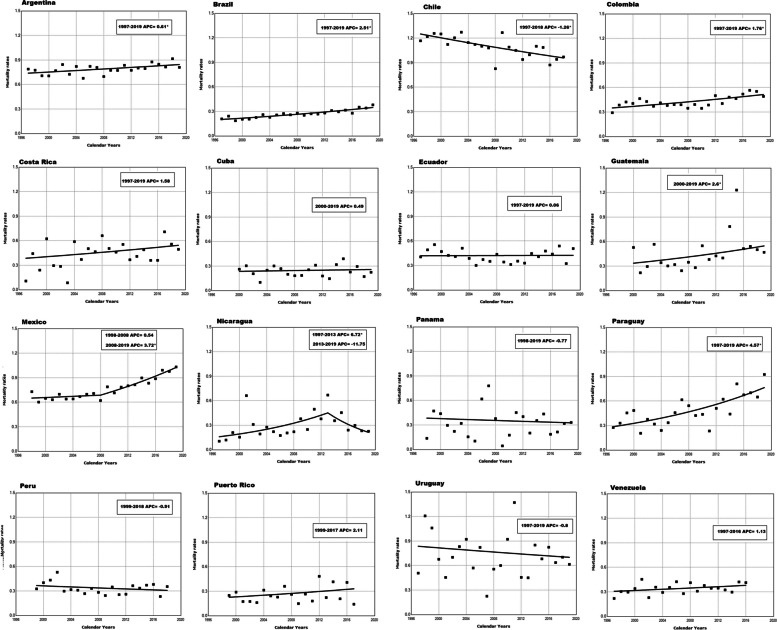
Annual percent change (APC) in testicular cancer mortality for all ages between 1997 and 2019

Table 1 shows the estimated AAPC in TC mortality for all ages between 1997 and 2019. Argentina (0.6%), Brazil (2.5%), Colombia (1.8%), Mexico (2.2%), and Paraguay (4.6%) had significant upward trends, whereas Chile (− 1.3%) showed a significant downward trend (Fig. 2).

**Table 1:** Average annual percent change and 95% confidence intervals for testicular cancer age-adjusted mortality rates in Latin American and the Caribbean for men of all ages, 1997–2019

Country	Age-Standardized Mortality Rates per 100 000		Trend 1	APC 1	Trend 2	APC 2	Average APC (95% CI)
	**1997**^**a**^	**2019**^**b**^					
Argentina	0.79	0.81	1997–2019	0.6* (0.2,1.0)			0.6* (0.2,1.0)
Brazil	0.21	0.38	1997–2019	2.5*(2.0,3.0)			2.5*(2.0,3.0)
Chile	1.17	0.97	1997–2018	− 1.3*(− 1.9, − 0.6)			− 1.3*(− 1.9, − 0.6)
Colombia	0.29	0.49	1997–2019	1.8*(1.0,2.6)			1.8*(1.0,2.6)
Costa Rica	0.11	0.50	1997–2019	1.6(− 0.7,3.9)			1.6(− 0.7,3.9)
Cuba	0.26	0.23	2000–2019	0.5(− 1.9,2.9)			0.5(− 1.9,2.9)
Ecuador	0.41	0.51	1997–2019	0.1(− 1.1,1.2)			0.1(− 1.1,1.2)
Guatemala	0.52	0.54	2000–2017	2.3(− 0.3,4.9)			2.3(− 0.3,4.9)
Mexico	0.73	1.03	1998–2008	0.5(− 0.8,1.9)	2008–2019	3.7*(2.8,4.7)	2.2*(1.4,3.0)
Nicaragua	0.10	0.23	1997–2013	6.7*(2.3,11.3)	2013–2019	− 11.7(− 23.6,1.9)	1.3(− 3.3,6.1)
Panama	0.14	0.33	1998–2019	− 0.8(− 4.3,3.0)			− 0.8(− 4.3,3.0)
Paraguay	0.28	0.93	1997–2019	4.6*(2.8,6.4)			4.6*(2.8,6.4)
Peru	0.33	0.35	1999–2018	− 0.9(− 2.5,0.7)			− 0.9(− 2.5,0.7)
Puerto Rico	0.25	0.14	1999–2017	2.1(− 1.1,5.4)			2.1(− 1.1,5.4)
Uruguay	0.51	0.61	1997–2019	− 0.8(− 3.0,1.5)			− 0.8(− 3.0,1.5)
Venezuela	0.22	0.41	1997–2016	1.1(− 0.3,2.6)			1.1(− 0.3,2.6)

*APC* Annual Percent Change, *CI* Confidence interval

^a^Data from 2000 for Cuba and Guatemala, 1998 for Mexico and Panama, 1999 for Peru and Puerto Rico

^b^Data from 2018 for Chile, 2017 for Guatemala, 2018 for Peru, 2017 for Puerto Rico, and 2016 for Venezuela; * *p*-value < 0.05

Table 2 presents the mortality rates and APCs for men in the age group 15–29 years between 1997 and 2019. Argentina (0.7%), Colombia (4.3%), Ecuador (3.5%), Guatemala (3.9%), Mexico (3.8%), Paraguay (4.8%), Peru (2.7%) and Venezuela (1.8%) showed significant upward trends for the entire time interval. Nicaragua also had a significant increasing trend from 1997–2013 (7.9), but the trend decreased from 2013–2019. Chile (− 1.4%) had a significant downward trend for the entire interval.

**Table 2:** Average annual percent change and 95% confidence intervals for testicular cancer age-adjusted mortality rates in Latin American and the Caribbean for men 15–29 years of age, 1997–2019

Country	Age-Standardized Mortality Rates per 100 000		Trend 1	APC 1	Trend 2	APC 2	Average APC (95% CI)
	**1997**^**a**^	**2019**^**b**^					
Argentina	1.24	1.39	1997–2019	0.7*(0.1,1.5)			0.7*(0.1,1.5)
Brazil	0.24	0.68	1997–2019	3.5*(2.7,4.3)			3.5*(2.7,4.3)
Chile	1.80	1.39	1997–2018	− 1.4*(− 2.5, − 0.3)			− 1.4*(− 2.5, − 0.3)
Colombia	0.44	0.82	1997–2019	4.3*(2.9,5.6)			4.3*(2.9,5.6)
Costa Rica	0.00	0.94	1999–2017	NA			NA
Cuba	0.30	0.24	2000–2017	NA			NA
Ecuador	0.48	0.71	1997–2019	3.5*(1.5,5.5)			3.5*(1.5,5.5)
Guatemala	0.67	0.86	2000–2019	3.9*(1.6,6.3)			3.9*(1.6,6.3)
Mexico	1.02	2.24	1998–2019	3.8*(3.3,4.2)			3.8*(3.3,4.2)
Nicaragua	0.42	0.20	1997–2013	7.9*(2.9,13.1)	2013–2019	− 14.0(− 4.9,8.2)	1.4(− 4.9,8.2)
Panama	0.47	0.35	1999–2019	NA			NA
Paraguay	0.29	1.28	1997–2019	4.8*(2.4,7.2)			4.8*(2.4,7.2)
Peru	0.42	0.49	1999–2018	2.7*(0.7,4.8)			2.7*(0.7,4.8)
Puerto Rico	0.47	0.00	1999–2017	NA			NA
Uruguay	0.99	1.28	1997–2019	− 1.6(− 4.7,1.6)			− 1.6(− 4.7,1.6)
Venezuela	0.41	0.61	1997–2016	1.8*(0.1, 3.6)			1.8*(0.1, 3.6)

*APC* Annual Percent Change, *CIs* Confidence intervals

^a^Data from 2000 for Cuba and Guatemala, 1998 for Mexico and Panama, 1999 for Peru and Puerto Rico

^b^Data from 2018 for Chile, 2017 for Guatemala, 2018 for Peru, 2017 for Puerto Rico, and 2016 for Venezuela; * *p*-value < 0.05;

Table 3 displays the mortality rates and APCs for men in the age group of 30–44 years between 1997 and 2019. Argentina (1.3%), Brazil (2.3%), Colombia (3.0%), and Mexico (3.2%) had significant increasing trends. In contrast, Chile (− 1.2%) and Peru (− 1.8%) presented significant downward trends.

**Table 3:** Average annual percent change and 95% confidence intervals for testicular cancer age-adjusted mortality rates in Latin American and the Caribbean for men 30–44 years of age, 1997–2019

Country	Age-Standardized Mortality Rates per 100 000		Trend 1	APC 1	Average APC (95% CI)
	**1997**^**a**^	**2019**^**b**^			
Argentina	1.24	1.39	1997–2019	1.3*(0.5,2.2)	1.3*(0.5,2.2)
Brazil	0.37	0.56	1997–2019	2.3*(1.4,3.2)	2.3*(1.4,3.2)
Chile	2.44	2.17	1997–2018	− 1.2*(− 2.2, − 0.2)	− 1.2*(− 2.2, − 0.2)
Colombia	0.45	0.78	1997–2019	3.0*(1.6,4.3)	3.0*(1.6,4.3)
Costa Rica	0.00	0.94	1999–2017	NA	NA
Cuba	0.30	0.24	2000–2017	NA	NA
Ecuador	0.26	0.67	1997–2019	0.1(− 2.0,2.3)	0.1(− 2.0,2.3)
Guatemala	0.47	0.74	2000–2019	2.8(− 0.6,6.2)	2.8(− 0.6,6.2)
Mexico	1.10	1.67	1998–2019	3.2*(2.4,4.1)	3.2*(2.4,4.1)
Nicaragua	0.42	0.20	1997–2019	NA	NA
Panama	0.47	0.35	1999–2019	NA	NA
Paraguay	0.29	1.28	1997–2019	NA	NA
Peru	0.63	0.50	1999–2018	− 1.8*(− 3.7,0.1)	− 1.8*(− 3.7,0.1)
Puerto Rico	0.47	0.00	1999–2017	NA	NA
Uruguay	0.59	0.61	1997–2019	− 2.2(− 5.4,1.2)	− 2.2(− 5.4,1.2)
Venezuela	0.24	0.71	1997–2016	1.4(− 0.7, 3.6)	1.4(− 0.7, 3.6)

*APC* Annual Percent Change, *CIs* Confidence intervals

^a^Data from 2000 for Cuba and Guatemala, 1998 for Mexico and Panama, 1999 for Peru and Puerto Rico

^b^Data from 2018 for Chile, 2017 for Guatemala, 2018 for Peru, 2017 for Puerto Rico, and 2016 for Venezuela; * *p*-value < 0.05

Table 4 shows the mortality rates and APCs for men aged ≥ 45 years between 1997 and 2019. For this age group, only Brazil (1.4%) presented a significant increase in mortality. In contrast, Colombia (− 1.9%), Ecuador (− 3.1%), Mexico (− 0.9%), and Peru (− 4.4%) the mortality rates significantly decreased.

**Table 4:** Average annual percent change and 95% confidence intervals for testicular cancer age-adjusted mortality rates in Latin American and the Caribbean for men ≥ 45 years of age, 1997–2019

Country	Age-Standardized Mortality Rates per 100,000		Trend 1	APC 1	Average APC (95% CI)
	**1997**^**a**^	**2019**^**b**^			
Argentina	0.94	0.79	1997–2019	− 0.4(− 1.3,0.5)	− 0.4(− 1.3,0.5)
Brazil	0.30	0.38	1997–2019	1.4*(0.5,2.3)	1.4*(0.5,2.3)
Chile	1.08	0.89	1997–2018	− 1.0(− 2.2,0.2)	− 1.0(− 2.2,0.2)
Colombia	0.40	0.54	1997–2019	− 1.9*(− 3.0, − 0.9)	− 1.9*(− 3.0, − 0.9)
Costa Rica	0.59	0.97	1999–2017	NA	NA
Cuba	0.40	0.24	2000–2019	0.4(− 2.7,3.6)	0.4(− 2.7,3.6)
Ecuador	0.92	0.70	1997–2019	− 3.1*(− 4.7, − 1.4)	− 3.1*(− 4.7, − 1.4)
Guatemala	0.96	0.42	2000–2019	2.7(− 2.3,7.9)	2.7(− 2.3,7.9)
Mexico	1.00	0.64	1998–2019	− 0.9*(− 1.8, − 0.1)	− 0.9*(− 1.8, − 0.1)
Nicaragua	0.37	0.00	1997–2019	NA	NA
Panama	0.64	0.35	1999–2017	NA	NA
Paraguay	0.85	0.51	1999–2017	NA	NA
Peru	0.37	0.52	1999–2018	− 4.4*(− 7.4, − 1.3)	− 4.4*(− 7.4, − 1.3)
Puerto Rico	0.11	0.13	1999–2017	NA	NA
Uruguay	0.59	0.71	1997–2019	1.6(− 1.1,4.4)	1.6(− 1.1,4.4)
Venezuela	0.25	0.51	1997–2016	− 0.2(− 3.4,3.2)	− 0.2(− 3.4,3.2)

*APC* Annual Percent Change, *CIs* Confidence intervals, *NA* Not applicable

^a^Data from 2000 for Cuba and Guatemala, 1998 for Mexico and Panama, 1999 for Peru and Puerto Rico

^b^Data from 2018 for Chile, 2017 for Guatemala, 2018 for Peru, 2017 for Puerto Rico, and 2016 for Venezuela; * *p*-value < 0.05

Supplementary table 1 displays the mortality rates and APCs for men aged 15–44 years. Nine countries showed significant increases in mortality rates: Argentina (1.0%), Brazil (3.0%), Colombia (3.9%), Ecuador (2.2%), Guatemala (3.6%), Mexico (3.6%), Nicaragua (3.8%), Paraguay (5.6%) and Venezuela (1.7%). In contrast, only Chile (− 1.3%) presented a significant decrease.

Regarding the evolution of incidence rates, Costa Rica and Quito (Ecuador) reported significant increases (around 3%) at all ages and of around 4.5% in the young population (15–29 years); in Colombia the only observed significant increase (of 4%) was observed in men aged 15 to 29 years (Supplementary table 2).

## Discussion

To our knowledge, this study on mortality by TC over a period of more than 20 years includes the largest of LAC countries to date. This study provides a comprehensive epidemiological analysis of TC mortality rates by age group in LAC between 1997 and 2019. Ten countries reported mortality rates higher than 0.43 per 100,000 men. We found a variation in mortality rates by age group and country. Overall, five countries (Argentina, Brazil, Colombia, Mexico, and Paraguay) presented significant increases in mortality, while Chile reported a significant decrease. Chile experienced significant decreases in rates among all men, except those aged ≥ 45 years of age, although the decline did not attain statistical significance. Four other countries (Colombia, Ecuador, Mexico, and Peru) showed significant decreases in TC mortality rates for men over 45 years of age.

Our findings of high TC mortality rates are consistent with previous studies describing the highest TC mortality in low-income countries and in the Central and South American regions [28]. The reasons for the increase in mortality by TC in Latin American countries are not clear. Some studies suggest a strong relationship between the human development index and TC. In fact, the incidence of TC has risen eightfold in developed regions compared to less developed countries [29]. However, no relationship has been found for mortality [28, 29]. Our study found significant increases in incidence rates in Cali (Colombia), Costa Rica, Quito (Ecuador) for the last few years reported. Despite this, the results for incidence were not the same for mortality in the countries mentioned. One of the important problems in Latin America and the Caribbean is the lack of incidence registry in most of the countries of the region, which would help to broaden the panorama of this disease [30]. In addition, data such as histology, time of disease, pathological antecedents, among others, would help to clarify the evolution of this disease in each country. On the other hand, changes in the quality of mortality data could explain the increases or decreases in the trends shown in our study [31].

Our study showed variations in mortality trends by age group, with an increase in the younger population, but, in some cases, a decrease in the population over 45 years of age. On the other hand, the increasing mortality rates in some countries suggest poor health care delivery or diagnosis at late stages of TC. Between 2003 and 2008, several Latin American countries (e.g., Brazil, Chile, and Mexico) enacted several laws to strengthen the health care systems towards universal health coverage. However, individuals at low socioeconomic levels have difficulty accessing prompt health care delivery [32]. As a result, in individuals in low economic settings the diagnosis of TC may be delayed and they may not receive optimal treatment, thereby increasing the mortality rate. For example, a study in men in the United States reported that uninsured men had a higher risk of advanced cancer and an 88% higher risk of germ cell cancer-specific mortality than those with health care insurance [33]. Similarly, a population-based study found that uninsured men with TC were more likely to present with metastatic disease and present higher mortality rates compared to insured men [34]. These observations highlight the need to perform early diagnosis and prompt treatment.

The increasing burden of cancer represents a substantial problem for LAC [35]; however, this represents only one facet of the problem, as the challenges in cancer care include other factors that condition cancer control. Among the existing challenges are insufficient financing, non-universal health coverage, inadequate registries, fragmented health systems, and unequal distribution of services, among others [36]. Although still lagging behind developed countries, according to The Lancet Oncology Commission 2015 report there have been improvements in cancer care in LAC, such as an increase in gross national product spent on cancer care and access to procedures and drugs, and basic health insurance that covers up to 60% of the population [37]. In regard to these changes, being a high-income country, Chile has responded with improvements in the management of cancer patients in recent years [37], with the creation of a “Acceso Universal con Garantias Explicitas” (apart from national or private insurance) that guarantees the treatment of all malignant neoplasms in children under 15 years of age and in 11 malignant diseases in adults [38]. These improvements in care could explain the decreasing mortality rate by TC found in the present study.

It should be taken into account that TC is a rare pathology and usually has a low associated mortality [39], and thus, the number of deaths is usually relatively small in all countries around the world. This could generate relatively unstable rates and make their analysis difficult, which may limit the quality of our results. The study also has several limitations inherent to the utilization of data from a secondary database, limited individual-level information, and variation in death registration completeness and quality. It is important to highlight the differences in the quality of mortality data records between countries and over time, which can lead to spurious trends and differences when comparing mortality rates. For this reason, it is important to meticulously evaluate completeness and quality of coding of mortality as well as promote high quality population-based caner registries and obtain better, more reliable information based on reliable data from the countries of the region. Nonetheless, a meticulous analysis of the information provided by the WHO database was carried out in order to provide the most accurate results.

On the other hand, this study has several strengths, such as lengthy study period (1997 to 2019) and the inclusion of a large number of countries across Latin America. In addition, the study examined mortality rates in various age groups to determine changes in rates in the different groups.

## Conclusion

In conclusion, our findings suggest geographical variability by age group in testicular cancer mortality rates between 1997 and 2019. The increasing testicular cancer mortality in the younger population is of concern and calls for early detection among symptomatic males and preventive interventions in the Latin American and the Caribbean population. It is important to improve the quality of data in our region, especially that related to incidence, in order to help broaden the picture of this neoplasm in Latin America.

### Supplementary Information

**Additional file 1: Supplementary table 1.** Average annual percent change and 95% confidence intervals for testicular cancer age-adjusted mortality rates in Latin American and the Caribbean for men 15-44 years of age, 1997-2019. 

**Additional file 2: Supplementary table 2.** Estimated annual percent change and confidence interval for testicular cancer incidence rates in Latin America and the Caribbean cancer registries and countries.

## Data Availability

The datasets generated and/or analyzed in the current study are available at the following link: https://www.who.int/data/data-collection-tools/who-mortality-database.
