# Supplementary material for: Testicular cancer mortality in Latin America and the Caribbean: trend analysis from 1997 to 2019
Source: BMC Cancer. 2023 Oct 27;23:1038. doi: 10.1186/s12885-023-11511-z (PMC10605564; doi:10.1186/s12885-023-11511-z)
Supplement: Supplementary file 1 — Additional file 1: Supplementary table 1. Average annual percent change and 95% confidence intervals for testicular cancer age-adjusted mortality rates in Latin American and the Caribbean for men 15-44 years of age, 1997-2019. [file 12885_2023_11511_MOESM1_ESM.docx]

Supplementary table 1. Average annual percent change and 95% confidence intervals for testicular cancer age-adjusted mortality rates in Latin American and the Caribbean for men 15-44 years of age, 1997-2019.

| **Country** | **Age-Standardized Mortality Rate per 100 000** | | **Trend 1** | **APC 1** | **Average APC (95% CI)** |
| --- | --- | --- | --- | --- | --- |
|  | **1997^a^** | **2019^b^** |  |  |  |
| Argentina | 1.22 | 1.39 | 1997-2019 | 1.0*(0.5,1.5) | 1.0*(0.5,1.5) |
| Brazil | 0.30 | 0.63 | 1997-2019 | 3.0*(2.5,3.6) | 3.0*(2.5,3.6) |
| Chile | 2.07 | 1.72 | 1997-2018 | −1.3*(−2.2,−0.5) | −1.3*(−2.2,−0.5) |
| Colombia | 0.39 | 0.80 | 1997-2019 | 3.9*(2.9,4.9) | 3.9*(2.9,4.9) |
| Costa Rica | 0.25 | 0.91 | 1999-2017 | 2.3(−0.6,5.3) | 2.3(−0.6,5.3) |
| Cuba | 0.29 | 0.38 | 2000-2019 | 1.9(−1.2,5.2) | 1.9(−1.2,5.2) |
| Ecuador | 0.39 | 0.70 | 1997-2019 | 2.2*(0.7,3.8) | 2.2*(0.7,3.8) |
| Guatemala | 0.59 | 0.81 | 2000-2019 | 3.6*(1.3,5.9) | 3.6*(1.3,5.9) |
| Mexico | 1.06 | 2.00 | 1998-2019 | 3.6*(3.1,4.1) | 3.6*(3.1,4.1) |
| Nicaragua | 0.24 | 0.24 | 1997-2019 | 3.8*(0.5,7.3) | 3.8*(0.5,7.3) |
| Panama | 0.32 | 0.61 | 1998-2019 | −1.2(−4.2,1.8) | −1.2(−4.2,1.8) |
| Paraguay | 0.46 | 1.35 | 1997-2019 | 5.6*(3.6,7.6) | 5.6*(3.6,7.6) |
| Peru | 0.51 | 0.49 | 1999-2018 | 0.6(−1.0,2.3) | 0.6(−1.0,2.3) |
| Puerto Rico | 0.38 | 0.27 | 1999-2017 | 0.6(−1.0,2.3) | NA |
| Uruguay | 0.59 | 0.71 | 1997-2019 | 2.3(−0.9,5.5) | 2.3(−0.9,5.5) |
| Venezuela | 0.34 | 0.65 | 1997-2016 | 1.7*(0.2,3.1) | 1.7*(0.2,3.1) |

^a^ Data from 2000 for Cuba and Guatemala, 1998 for Mexico and Panama, 1999 for Peru and Puerto Rico; ^b^ Data from 2018 for Chile, 2017 for Guatemala, 2018 for Peru, 2017 for Puerto Rico, and 2016 for Venezuela; * p-value < 0.05;

APC, Annual Percent Change; CIs, confidence interval; NA: not applicable
