# Supplementary material for: Testicular cancer mortality in Latin America and the Caribbean: trend analysis from 1997 to 2019
Source: BMC Cancer. 2023 Oct 27;23:1038. doi: 10.1186/s12885-023-11511-z (PMC10605564; doi:10.1186/s12885-023-11511-z)
Supplement: Supplementary file 2 — Additional file 2: Supplementary table 2. Estimated annual percent change and confidence interval for testicular cancer incidence rates in Latin America and the Caribbean cancer registries and countries. [file 12885_2023_11511_MOESM2_ESM.docx]

Supplementary table 2. Estimated annual percent change and confidence interval for testicular cancer incidence rates in Latin America and the Caribbean cancer registries and countries.

| **Country/Registry** | **Period** | **All ages combined** | **15−29** | **30-44** | **45+** |
| --- | --- | --- | --- | --- | --- |
| **Incidence** |  |  |  |  |  |
| Brazil, Goiania | 1999−2012 | 1.0(−4.6,6.8) | 1.2(−6.6,9.7) | NA | NA |
| Chile, Valdivia | 1999−2012 | 1.0(−2.1,4.1) | 0.8(−3.9,5.6) | 2.2(−4.6,9.4) | 0.2(−7.5,8.5) |
| Colombia, Cali | 1999−2012 | 2.2(−1.1,5.7) | 4.0*(0.4,7.8) | 2.9(−2.8,8.9) | −2.5(−11.2,7.2) |
| Costa Rica | 1999−2011 | 3.3*(1.1,5.5) | 4.3*(2.1,6.6) | 2.9(−0.3,6.2) | −0.5(−6.2,5.6) |
| Ecuador, Quito | 1999−2012 | 3.1*(0.4,5.8) | 4.8*(0.4,9.5) | 1.5(−2.1,5.1) | 1.1(−5.9,8.6) |

NA: not applicable
